# ROBO1 is required for postnatal growth and survival

**DOI:** 10.1007/s13258-026-01802-7

**Published:** 2026-09-01

**Authors:** Nicole Aurora Kirk, Ye Eun Hwang, Seul Gi Lee, Juyeon Kang, Kate-Lin Ly, Yong Yu Gan, Sangmyung Rhee, Kee-Beom Kim, Kwon-Sik Park

**Affiliations:** 1 Department of Microbiology, Immunology, and Cancer Biology, University of Virginia School of Medicine, 1340 Jefferson Park Avenue, Charlottesville, VA 22903 USA; 2 Department of Life Sciences, Chung-Ang University, Seoul, Republic of Korea; 3 BK21 FOUR KNU Creative BioResearch Group, Kyungpook National University, Daegu, Republic of Korea

**Keywords:** ROBO1, Growth hormone, Insulin-like growth factor 1

## Abstract

Roundabout guidance receptor 1 (ROBO1) is critical for various processes essential to mammalian development. However, its role in postnatal development and growth remains poorly understood, primarily due to perinatal lethality in knockout models. *Robo1* knockout mice on a mixed B6/129S genetic background survive into adulthood and exhibit previously unreported abnormalities across multiple organs. These mice display stunted growth and organ-specific defects, including enlarged lung alveoli, premature hair graying, and diminished subcutaneous fat. Notably, these mice have a reduced lifespan. At 23 days of age, corresponding to the beginning of early puberty in mice, *Robo1* knockout mice show significantly reduced plasma levels of growth hormone (GH) and insulin-like growth factor 1 (IGF-1), paralleling decreased expression in the pituitary gland and liver, respectively, compared to wild-type littermates. These findings suggest that ROBO1 contributes to the postnatal development and functional maintenance of multiple organs, with associated modulation of the GH/IGF-1 axis, potentially influencing survival. Further studies with larger cohorts are warranted to elucidate the broader implications of ROBO1 deficiency, particularly in relation to human developmental disorders such as growth hormone deficiencies.

**Supplementary Information:**

The online version contains supplementary material available at https://doi.org/10.1007/s13258-026-01802-7.

## Highlights

Germline loss of ROBO1 results in stunted postnatal growth and reduced survival in mice.

ROBO1-deficient mice undergo aberrant development of various organs, including pituitary gland, skin, and lungs.

ROBO1 loss impairs the GH/IGF-1 axis during early puberty in mice.

**Supplementary Information:**

The online version contains supplementary material available at https://doi.org/10.1007/s13258-026-01802-7.

## Introduction

ROBO1, a transmembrane receptor of the immunoglobulin superfamily, is highly conserved across species and plays critical roles in various developmental processes (Kidd et al. 1998). It is best known for guiding axonal branching, pathfinding, and migration in the nervous system (Fujiwara et al. 2006; Kidd et al. 1998). Mutations in *ROBO1* disrupt axonal guidance during brain development, leading to aberrant midline crossing of growth cones (Andrews et al. 2006; López-Bendito et al. 2007; Seeger et al. 1993), and are associated with neurological disorders such as pituitary stalk interruption syndrome (PSIS), combined pituitary hormone deficiency (CPHD), and dyslexia (Bashamboo et al. 2017; Hannula-Jouppi et al. 2005; Xu et al. 2015).

Emerging evidence suggests a role for ROBO1 in non-neural organ development. *Robo1* knockout mouse embryos exhibit cardiac malformations, including pericardial and ventricular septal abnormalities, although these phenotypes are limited to the prenatal period (Kruszka et al. 2017; Mommersteeg et al. 2013). Mice carrying ENU-induced homozygous missense mutations (*Robo1*^Ile270Thr/Ile270Thr^) survive to birth, presenting only craniofacial and urinary tract anomalies (Kruszka et al. 2017; Münch et al. 2022). Thus, the broader postnatal impact of ROBO1 deficiency on organ development remains poorly understood, largely due to the lack of appropriate animal models.

## Materials and Methods

### Genes

**Table:** 

Name	ID	Size	Chromosome location	ENSMBL ID
Roundabout guidance receptor 1	*Robo1*	737,790 bases	Chromosome 16: 72,105,194–72,842,983	ENSMUSG00000022883
Growth hormone	*Gh*	1595 bases	Chromosome 11: 106,191,097–106,192,691	ENSMUSG00000020713
Insulin like growth factor 1	*Igf1*	78,778 bases	Chromosome 10: 87,694,127–87,772,904	ENSMUSG00000020053

#### Mice

The generation of *Robo1* knockout allele in mice was previously described (Long et al. 2004). *Robo1* +/– mice carrying heterozygous *Robo1* knockout allele were crossed with in-house strains of mice with a mixed B6/129S background. Animal procedures for this study adhered to the guidelines from the National Institutes of Health in the United States and were approved by the Institutional Animal Care and Use Committee at the University of Virginia. Tails of pups from the crosses of male and female *Robo1* +/– mice were collected before weaning and were digested in lysis buffer containing proteinase K (Thermo Fisher Scientific cat# BP1700-100). DNAs were purified using potassium acetate and subsequently in 50% ethanol, resuspended in water, and added to a polymerase chain reaction (PCR) mix containing the following primers: forward primer common to both wild-type and mutant alleles 5′-TGGCACGAAGGTATATGTGC-3′, a wild-type allele-specific reverse primer 5′-GAAGGACTGGTGGTTTTGAG-3′, and a mutant allele-specific reverse primer 5′-CCTCCGCAAACTCCTATTTC-3′ (Long et al. 2004). The PCR was performed at 95℃ for 5 min; 30 cycles of 95℃ for 20 s, 58℃ for 45 s, and 72℃ for 45 s; finally, 72℃ for 10 min. The PCR products were separated in 3% agarose gel and were visualized under UV light. Body length was the greatest straight-line distance from the top of the head (crown) to the bottom of the buttock (rump). Mouse growth curves and Kaplan–Meier survival plots display changes in animal at 4-week intervals starting at 3 weeks of age.

### Histology and immunoblot analysis

Various organs, including the brain, lung, liver, and skin, were collected from euthanized mice and were fixed in 4% paraformaldehyde in phosphate-buffered saline (PBS) at 4 ℃ overnight before being processed for paraffin embedding. Five-micron sections of paraffin blocks were stained with hematoxylin and eosin (H&E). Fontana-Masson stain was performed to visualize melanin deposit in the skin sections, using a Fontana-Masson kit (MilliporeSigma catalog# HT2000). Light microscopy images of skin sections stained with H&E or Fontana-Masson were obtained using Nikon Eclipse Ni-U microscope (Nikon). Image analysis and automated quantification of target parameters were performed using NIS-Elements Basic Research software (Nikon). To examine morphology of alveolar sacs, 20× magnified images of the peripheral lungs taken of the H&E-stained lung sections. Five of the 20× images were acquired from each lung section and were analyzed using CellProfiler software. The number of alveolar sacs was analyzed using the “IdentifyPrimaryObject” pipeline. Mean area and perimeter were analyzed using the “MeasureObjectSize” pipeline.

To perform immunoblot analysis, tissues were lysed in RIPA buffer (50 mM Tris–HCl pH7.4, 150 mM NaCl, 2 mM EDTA, 1% NP-40, 0.1% SDS). 10 μg protein lysate was loaded into various percentages of SDS polyacrylamide gels for electrophoresis in PAGE running buffer (25 mM Tris, 192 mM glycine, 0.1% SDS, pH8.3). Proteins were then transferred to 0.2 or 0.45 μm PVDF membrane in transfer buffer (25 mM Tris, 192 mM glycine, 20% methanol). Membranes were blocked for 1 h at room temperature using TBST buffer (150 mM NaCl, 10 mM Tris pH8.0, 0.1% Tween20) supplemented with 3% non-fat milk, then blotted with primary antibodies overnight at 4 °C on an orbital shaker. After washing 5 times with TBST, membranes were incubated with secondary antibodies for 2 h at room temperature and washed another 5 times with TBST. Membranes were then incubated with ECL Western Blotting Detection Reagent (Thermo Fisher Scientific catalog# 32,106) or ECL Substrate Plus Kit (BioLegend catalog# 426,315) for 5 min, and signals were detected using a ChemiDoc machine (Bio Rad). The primary antibodies and dilutions are mouse anti ACTB (Santa Cruz Biotechnology, catalog# sc-47778, 1:5000), rabbit anti-GH (Thermo Fisher Scientific catalog# GH-101AP, 1:750), rabbit anti-IGF-1 (Cell Signaling Technology, catalog# E6B70, 1:750). The secondary antibodies and dilutions are goat anti mouse IgG (Jackson ImmunoResearch catalog# 115–035-003, 1:5000) and goat anti-rabbit IgG (Jackson ImmunoResearch catalog# 111–035-003, 1:5000).

### Plasma collection and enzyme linked immunosorbent assays

Littermates of mice at 23 days and 2 months of age were euthanized using a sublethal dose of avertin injection and blood (200 μL) from the retro-orbital sinus was collected in K2-EDTA microtainer tubes (BD Bioscience 365,974). Plasma was separated by centrifugation at 1500 × g for 15 min using a refrigerated tabletop centrifuge, immediately frozen using dry ice, and stored at − 80℃. To quantify levels of GH and IGF-1, the frozen plasma samples were thawed on ice and subjected to enzyme linked immunosorbent assay (ELISA) using commercial kits (AFG Scientific catalog EK247312 and Proteintech catalog KE10032, respectively) using the manufacturer protocols (www.afgsci.com/pdf/sandwich.php?product_id=653018; www.ptglab.com/products/pictures/pdf/Mouse-IGF1-ELISA-Kit-KE10032-datasheet.pdf). Briefly, the plasma samples were diluted in sample diluent (1% bovine serum albumin, 0.05% Tween-20 in PBS) at 1:5 for GH ELISA and 1:300 for IGF-1 ELISA and stored at − 20℃ until the assay was performed. Processed samples in the ELISA were read at 450 nm with the correction wavelength set at 650 nm using spectrophotometer (BioTek). The sensitivity of GH ELISA is 34 pg/mL and the detection range is 78.13–5000 pg/mL and the sensitivity of IGF-1 ELISA is 1.3 pg/mL and the detection range is 15.6–1000 pg/mL. Both GH and IGF-1 are known to have very short half-lives (4–20 min for GH, 10–15 min for free IGF-1, and approximately 12 h for the IGFBP3-bound form of IGF-1).

### Statistical analysis

Statistical analysis and drawing graphs were done using GraphPad Prism 9.0 (GraphPad Software). Unpaired student t-tests were used for the comparison of two groups. *Robo1* wild-type vs. mutant mice. Sample size per group to achieve 95% power was calculated using pilot data (e.g. mean body lengths: 9 cm for wild-type mice and 7.5 cm for mutant mice. standard deviation 0.5 cm; mean body weight; mean body weights are 21 g for wild-type mice and 11 g for mutant mice. standard deviation 4.0 g). One-way Analysis of Variance (ANOVA) was used for comparisons of more than two groups. Kaplan–Meier curves were used for plotting mice survival and log-rank test was used to determine significance with *p*<0.05 considered to be significant. Results are presented as the mean ± standard deviation (SD) and statistical significance was set as *p* <0.05 (*p<0.05, **p<0.01, and ***p<0.001).

## Results and discussion

**Fig. 1:**
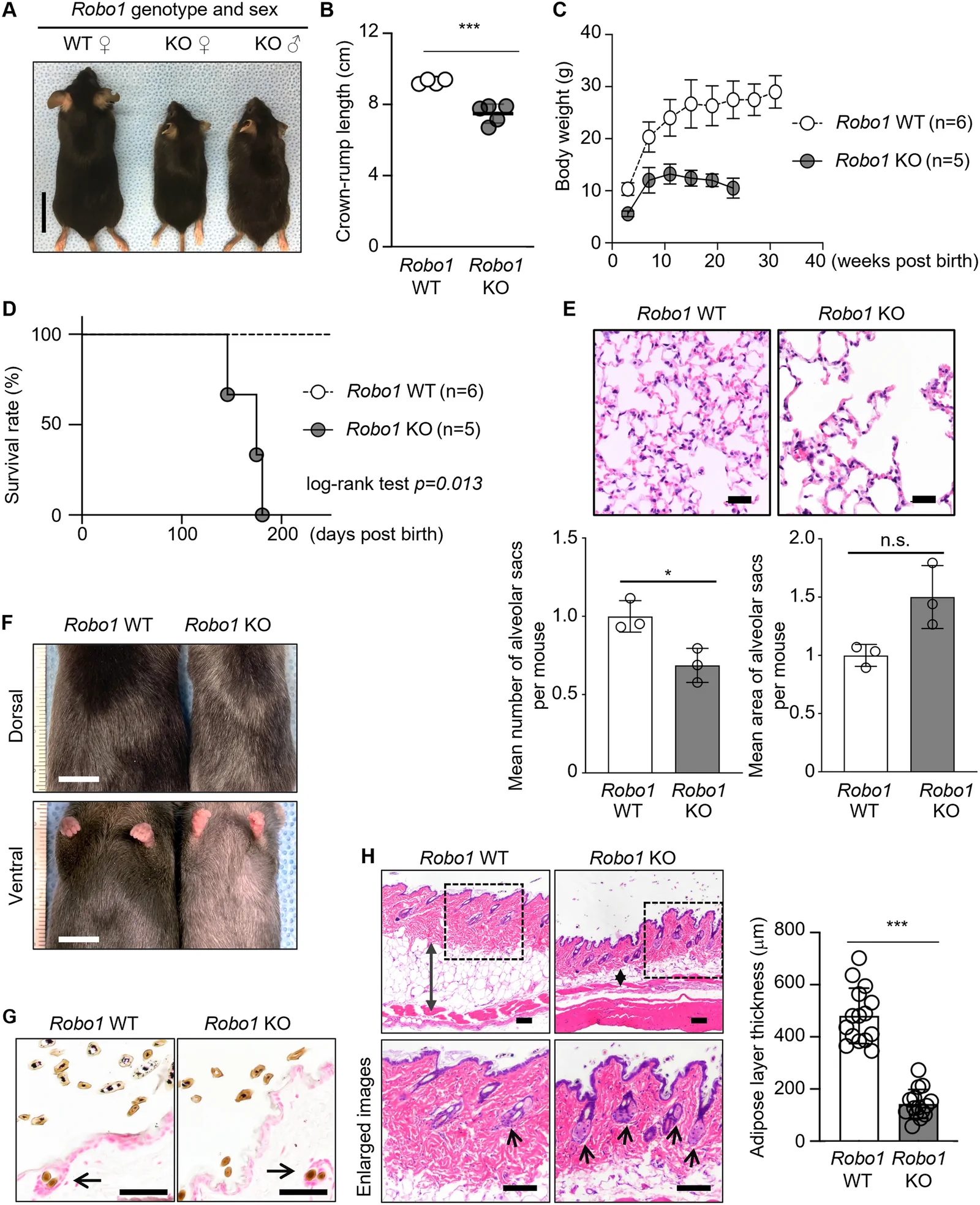
Postnatal growth defects and shortened life span in ROBO1 deficient mice. **A** A representative image showing the dorsal side of *Robo1* WT and KO mice at 2 months of age. Of note, both male and female *Robo1* KO mice were much smaller than the female Robo1 *WT*. **B** Plot of crown-rump length of *Robo1* WT (n=4 mice) and KO (n=5 mice) at 7 weeks of age.** C** Plot of body weight monitored at 4-week intervals starting at 3 weeks of age.** D** Kaplan–Meier survival curves of *Robo1* WT (n=6 mice) and KO (n=5 mice).** E** Top, Representative images of H&E-stained sections of peripheral lungs of *Robo1* WT and KO mice at 2 months of age. Bottom, plots for relative mean number of alveolar sacs per mouse and relative mean area of alveolar sacs per mouse in the H&E images. Each point represents the average of 5 ROIs per mouse (n=3 mice per genotype).** F** Representative pictures of *Robo1* WT and KO mice at 10 weeks of age. The hairs in *Robo1* KO mice are grayer than those in control littermates.** G** Representative images of Fontana-Masson-stained skins in mice. Arrows indicate the staining in hair shafts.** H** Left, Representative images of H&E-stained sections of skin of *Robo1* WT and KO mice. Double arrows indicate the lengths of subcutaneous fat layers. Arrows indicate sebaceous glands. Right, Plot of adipose layer thickness in *Robo1* WT and KO mice. Results are an average of 3 lines per section, and 5 sections per mouse (n=3 mice per group). ***p < 0.001. ****p < 0.0001. Data are presented as the mean ± standard deviation (SD) in H. Statistical analyses were performed using unpaired t-test (**B**,** E**,** H**) and log-rank (Mantel-Cox) test (**C**). Scale bars: 10 μm (**A**); 1 cm (**F**); 10 cm (**G**); 50 μm (**H**)

To determine the postnatal impact of *Robo1* loss, we crossed male and female *Robo1*± mice. Genotyping PCR analysis of pups at 23 days of age showed that each litter from the genetic cross generally yielded a 1:2:1 ratio of *Robo1*+/+, *Robo1*+/–, and *Robo1*–/– genotypes (Supplemental Fig. 1A), confirming that pups of all genotypes were born alive and survived the postnatal period. This finding was somewhat unexpected given previous reports that complete deletion of *Robo1* was perinatally lethal in the B6 genetic background (Andrews et al. 2006). We did not observe overt differences in body size among newborns; differences gradually became apparent, with body weight of *Robo1* KO mice significantly reduced by 3 weeks of age. The mixed B6/129S genetic background may have mitigated the prenatal defects and perinatal lethality reported in pure B6 mice, consistent with the influence of genetic background on the severity of *Robo1* knockout phenotypes. Notably, both male and female *Robo1*–/– mice were much smaller than *Robo1*+/– and +/+ littermates, regardless of sex (Fig. 1A). *Robo1*+/+ and *Robo1*+/– mice were similar in size and were therefore designated as *Robo1* WT, whereas *Robo1*–/– mice were designated as *Robo1* KO.

At 7 weeks of age, the body length (the crown-rump length) and weight of *Robo1* KO mice were significantly lower than those of *Robo1* WT mice (Fig. 1B and Supplemental Fig. 1B). To determine whether this reflected a simple delay in growth, we monitored cohorts of mice at 4-week intervals starting at 3 weeks of age. In mice, postnatal development includes a period of rapid growth between weeks 3 and 7, corresponding to puberty. At 3 weeks of age, both the weight and growth rate of *Robo1* KO mice were already significantly lower than *Robo1* WT controls. The difference in body weight between groups widened over time (Fig. 1C). Despite surviving through the typical growth period, *Robo1* KO mice began dying as early as 51 days of age, with a median survival age of 172 days. All *Robo1* WT littermates survived to the end of experiments (Fig. 1D). Beginning at 2 months of age, *Robo1* KO mice gradually became less active and their movement slowed, whereas *Robo1* WT littermates remained active, as expected (not shown). Together, these results indicate that ROBO1 deficiency causes growth defects and shortens lifespan in mice.

Multiple factors may underlie the observed phenotypes. In addition to reduced size and weight, several organs in *Robo1* KO mice appeared abnormal, including the lungs and skin. Morphometric analysis of the lung parenchyma revealed a significant reduction in alveolar sac number in *Robo1* KO mice compared to *Robo1* WT littermates. In line with this, the relative area of the alveolar sacs was increased in *Robo1* KO mice, although this difference was not statistically significant (Fig. 1E). No other morphological differences were noted in the bronchioles or airway epithelium (Supplemental Fig. 1C). These alveolar changes are consistent with previous reports that endoderm-selective *Robo1* deletion significantly reduces gas-exchange surface area (Branchfield et al. 2016) and that germline *Robo1* knockout can result in neonatal lethality, labored breathing, and altered lung morphology (Xian et al. 2001). These findings suggest that the lung abnormality arising in postnatal development persists into adulthood. Other remarkable phenotypes include the appearance of gray skin hair at a very early age in *Robo1* KO mice, most prominently on the ventral side (Fig. 1F). Fontana-Masson stain revealed reduced melanin in hair shafts of *Robo1* KO mice compared to *Robo1* WT littermates (Fig. 1G), although epidermal melanin levels were similar between groups. H&E staining of skin showed that *Robo1* KO mice had a significantly thinner subcutaneous fat layer and enlarged sebaceous glands compared to WT controls (Fig. 1H).

**Fig. 2:**
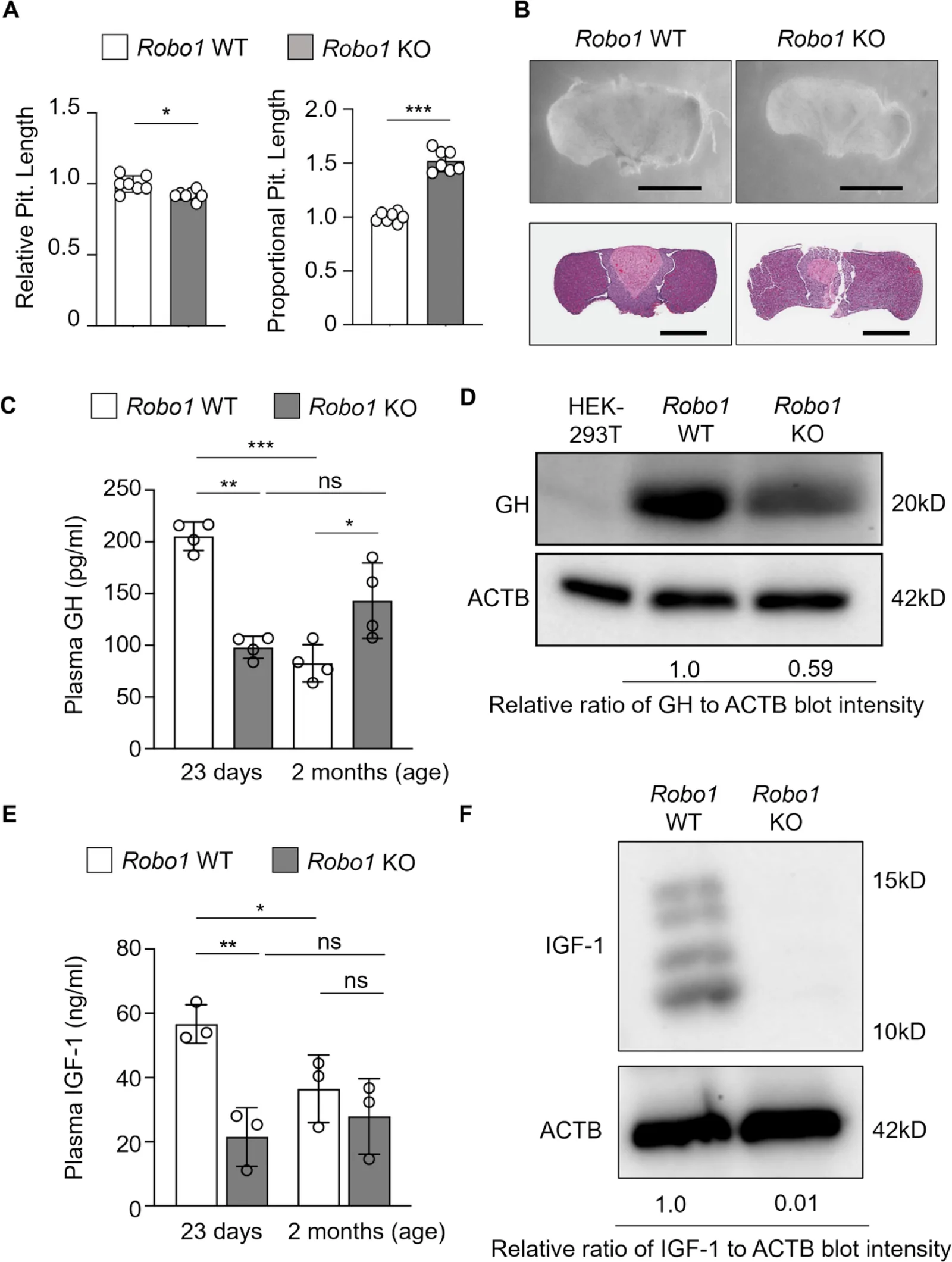
Reduced levels GH and IGF-1 in both plasma and tissues of ROBO1 deficient mice.** A** Left, relative absolute length of the pituitary glands of *Robo1* WT and KO mice (n=7 mice per genotype). Right, relative size of the pituitary gland proportional to body weight in *Robo1* WT and KO mice (n=7 mice per genotype).** B** Top, representative whole-mount images of pituitary glands dissected from *Robo1* WT and KO mice at 23 days of age. Bottom, representative images of H&E-stained sections of the pituitary glands in mice at 23 days of age. **C** Plot of GH concentrations in the plasma of *Robo1* WT and KO mice at 23 days and 2 months of age. Data shown as mean ± SD from 4 mice per genotype, across 3 independent experiments.** D** Immunoblot of GH in lysates from the pituitary glands of *Robo1* WT and KO mice at 23 days of age with lysate from HEK293T cells as a negative control.** E** Plot of IGF-1 concentrations in the plasma of *Robo1* WT and KO mice at 23 days and 2 months of age. Data shown as mean ± SD from 3 mice per genotype, across 3 independent experiments** F** Immunoblot of IGF-1 in the lysates of *Robo1* WT and KO livers at 23 days of age. *p < 0.05, **p < 0.01, ***p < 0.001, ns not significant. Statistical analyses were performed using ANOVA (**C**,** E**) and unpaired t-test (**A**). Scale bars: 500 μm (**B**).

The diversity of phenotypes in *Robo1* KO mice suggests a systemic role for ROBO1 in postnatal growth and survival. The growth hormone (GH)/insulin-like growth factor 1 (IGF-1) axis is a central regulator of development and growth (Bashamboo et al. 2017). GH, secreted from the pituitary gland, stimulates IGF-1 production to promote growth (Schneider et al. 2003). Both hormones peak during puberty, driving skeletal growth and nutrient utilization. Disruption of GH or IGF-1 production causes severe developmental abnormalities (Poudel et al. 2020). Consistent with reports of smaller pituitary size in patients with *ROBO1* mutation (Bar et al. 2015; Bashamboo et al. 2017; Liu and Chen 2020), *Robo1* KO mice had smaller pituitary glands than WT littermates; however, pituitary size was proportionally larger relative to body weight in mice (Fig. 2A). This pattern suggests that ROBO1 deficiency does not directly reduce the size of the pituitary gland and that the absolute size difference is secondary to the disproportionate reduction in overall body mass. Histological analyses revealed no overt differences between genotypes (Fig. 2B). To investigate whether short stature in *Robo1* KO mice was linked to GH deficiency, we measured plasma GH levels at 23 days and 2 months of age. At 23 days, *Robo1* KO mice had significantly lower GH levels than WT mice, while at 2 months, levels were modestly higher (Fig. 2C). Consistent with the patterns of plasma GH levels, immunoblot analysis of pituitary lysates confirmed markedly reduced GH content in 23-day-old *Robo1* KO mice (Fig. 2D, Supplemental Fig. 2A).

Because GH exerts its growth-promoting effects by stimulating hepatic IGF-1 production and its release into the bloodstream (Münch et al. 2022), we measured IGF-1 levels in the same mice. At 23 days, plasma IGF-1 levels were significantly reduced in *Robo1* KO mice compared to WT controls, whereas no significant differences were seen at 2 months (Fig. 2E). Liver lysates from 23-day-old KO mice also contained markedly less IGF-1 compared to WT controls (Fig. 2F, Supplemental Fig. 2B). These results suggest that transient suppression of GH/IGF-1 during puberty is associated with long-term growth impairment and premature death. It should be noted, however, that the growth deficit in *Robo1* KO mice was already apparent at 3 weeks of age (prior to peak pubertal GH secretion), suggesting that ROBO1-dependent mechanisms during early postnatal development may also contribute to growth restriction. Moreover, we cannot exclude the possibility that the premature aging phenotype observed in *Robo1* KO mice secondarily suppresses GH/IGF-1 levels, as aging is a known modulator of the somatotropic axis (Poudel et al. 2020). Resolving the precise causal sequence between ROBO1 loss, reduced GH/IGF-1, and premature aging will require future studies, ideally with conditional knockout models and larger, sex-stratified cohorts. Despite reduced IGF-1, liver histology appeared normal, and other examined organs, including the kidneys, showed no gross abnormalities (Supplemental Fig. 3A, B) (Niu et al. 2013; Kamenický et al. 2014; Park and Jin 2023).

This study demonstrates that *Robo1* KO mice can survive into adulthood, likely aided by their mixed genetic background (B6/129S), which may compensate for ROBO1 loss. This new model of ROBO1 deficiency reveals previously unreported phenotypes, including severe postnatal growth defects, early mortality, progressive motor decline, alveolar enlargement, premature hair graying, and a reduced subcutaneous fat layer. Consistent with these defects, *Robo1* KO mice had significantly reduced plasma levels of GH and IGF-1 at weaning. Together, the data indicate that the ROBO1–GH–IGF-1 axis is a key regulator of postnatal growth and survival.

The observed phenotypes have potential clinical relevance. In humans, *ROBO1* mutations are linked to pituitary stalk interruption syndrome (PSIS) and combined pituitary hormone deficiency (CPHD) (Bar et al. 2015; Bashamboo et al. 2017; Liu and Chen 2020). These congenital disorders feature GH deficiency, reduced body weight, and delayed growth, primarily due to pituitary dwarfism and impaired hormone secretion. While inactivating mutations in *POU1F1*, *PROP1*, and *PTCH1* are associated with PSIS and CPHD (Brauner et al. 2020; Diwaker et al. 2022), this study suggests *ROBO1* inactivation as part of the molecular mechanisms underlying the pathological conditions. In addition, reduced expression of ROBO1 signaling pathway components has been reported in patients with chronic obstructive pulmonary disease (COPD) (Lin et al. 2019). In that study, cigarette smoke exposure induced emphysema and was associated with decreased *ROBO1* transcription. In mouse lung epithelial cells, it was found that decreased SLIT2/ROBO1 interactions reduced the migration and wound healing capabilities of the cells and further hindered lung regeneration in an emphysema model (Park et al. 2021). In our model, *ROBO1* loss alone was sufficient to cause alveolar sac enlargement—a hallmark of emphysema—highlighting the potential role of *ROBO1* in lung development and disease. These findings provide functional evidence that *ROBO1* deficiency may contribute to the etiology of human disorders.

Several limitations of this study should be noted. The relatively small number of mice were analyzed, and results were not stratified by sex. Although *Robo1* KO mice, regardless of sex, were consistently smaller than their *Robo1* WT littermates, the impact of GH/IGF-1 on growth can be sexually dimorphic. Therefore, future studies with larger cohorts are needed to draw more definitive conclusions regarding the systemic and sex-specific effects of ROBO1 loss. The role of ROBO1 in organ development and in regulating GH/IGF-1 expression remains unclear. Similarly, the expression and activity of GHR/IGF1R or downstream JAK2/STAT5 signaling were not assessed. Additional studies are warranted to establish a comprehensive mechanistic link between ROBO1 and the growth factor signaling axis. Functional studies such as recombinant GH supplementation to assess whether growth deficits can be rescued would help clarify the causal contribution of reduced GH/IGF-1 signaling. Future studies employing immunohistochemistry or in situ hybridization to enumerate hormone-producing cell populations in the pituitary gland, as well as immunoblots displaying multiple individual biological replicates side by side, would strengthen these findings. Similarly, measuring levels of additional pituitary-derived hormones (e.g., ACTH, PRL, FSH) and metabolic parameters (e.g., fasting glucose and insulin) would provide a more complete picture of the endocrine consequences of ROBO1 deficiency. Further characterization of the premature aging phenotype through a comprehensive assessment of established aging markers will be pursued; these studies are planned in a reconstituted mouse cohort.

In conclusion, despite these limitations, this study demonstrates that ROBO1 deficiency during fetal development leads to significant physiological consequences beyond perinatal death. The results suggest that ROBO1 plays a novel role in regulating somatotropic growth during early puberty, influencing the development and function of multiple organs, and ultimately impacting long-term survival. Furthermore, *Robo1* KO mice provide a valuable model for investigating human disorders such as PSIS and CPHD, and for exploring potential therapeutic strategies.

## Supplementary Information

Below is the link to the electronic supplementary material.

Supplementary Material 1

## Data Availability

The supporting data including images can be assessed directly from the corresponding author upon request.
