# Supplementary material for: ROBO1 is required for postnatal growth and survival
Source: Genes Genomics. 2026 Sep 1;48(10):1265–73. doi: 10.1007/s13258-026-01802-7 (PMC13661687; doi:10.1007/s13258-026-01802-7)
Supplement: Supplementary file 1 [file 13258_2026_1802_MOESM1_ESM.docx]

**Supplemental Figure 1. Differences in weight and lung morphology *Robo1* WT and KO mice.** (A) A representative gel electrophoresis image showing results of Robo1 genotyping PCR. (B) Plot of body weight of *Robo1* WT (n=7 mice per genotype) and KO (n=8 mice per genotype) mice at 7 weeks of age. (C) Representative images of H&E-stained sections of peripheral lungs of *Robo1* WT and KO mice at 2 months of age. Data is presented as the mean ± SD. Statistical analysis was performed using unpaired t-test. ****p* < 0.001. Scale bars: 100μm.

**Supplemental Figure 2. Reduced levels GH and IGF-1 in tissues of ROBO1 deficient mice.** Technical replicates of the immunoblots shown in Figure 2. (A) Immunoblot of GH in lysates from the pituitary glands of *Robo1* WT and KO mice at 23 days of age with lysate from HEK293T cells as a negative control. (B) Immunoblot of IGF-1 in the lysates of *Robo1* WT and KO livers at 23 days of age.

**Supplemental Figure 3. Comparison of multiple organs between *Robo1* WT and KO mice.**  (A) Representative images of H&E-stained sections of the livers of *Robo1* WT and KO mice at 2 months of age. (B) Representative images of H&E-stained sections of the kidney, pancreas, and colon of *Robo1* WT and KO mice at 2 months of age. Scale bars: 10μm (A, B).
